# Design, Synthesis and Evaluation of 3-(2-Aminoheterocycle)-4-benzyloxyphenylbenzamide Derivatives as BACE-1 Inhibitors

**DOI:** 10.3390/molecules18033577

**Published:** 2013-03-20

**Authors:** Shihao Shangguan, Fei Wang, Yong Liao, Haiping Yu, Jia Li, Wenhai Huang, Haihong Hu, Lushan Yu, Yongzhou Hu, Rong Sheng

**Affiliations:** 1 ZJU-ENS Joint Laboratory of Medicinal Chemistry, College of Pharmaceutical Sciences, Zhejiang University, Hangzhou 310058, China; 2 The National Center for Drug Screening, Shanghai 201203, China; 3 Institute of Materia Medica, Zhejiang Academy of Medical Sciences, Hangzhou 310013, China; 4 Department of Pharmaceutical Analysis and Drug Metabolism, College of Pharmaceutical Sciences, Zhejiang University, Hangzhou 310058, China

**Keywords:** BACE-1 inhibitor, 2-amino-6*H*-1,3,4-thiadizine, blood-brain barrier permeability

## Abstract

Three series of 3-(2-aminoheterocycle)-4-benzyloxyphenylbenzamide derivatives, 2-aminooxazoles, 2-aminothiazoles, and 2-amino-6*H*-1,3,4-thiadizines were designed, synthesized and evaluated as *β*-secretase (BACE-1) inhibitors. Preliminary structure-activity relationships revealed that the existence of a 2-amino-6*H*-1,3,4-thiadizine moiety and α-naphthyl group were favorable for BACE-1 inhibition. Among the synthesized compounds, **5e** exhibited the most potent BACE-1 inhibitory activity, with an IC_50_ value of 9.9 μΜ and it exhibited high brain uptake potential in Madin-Darby anine kidney cell lines (MDCK) and a Madin-Darby canine kidney-multidrug resistance 1 (MDCK-MDR1) model.

## 1. Introduction

More than 25 million people in the world are suffering from dementia, the most common form among which is Alzheimer’s Disease (AD), characterized by progressive memory loss and cognitive decline [1]. The major pathological hallmark for AD research is the unfolding of amyloid plaques and neurofibrillary tangles. The amyloid cascade hypothesis, which stated that accumulation of β-amyloid (Aβ) in the brain is the leading factor in the pathogenesis of Alzheimer’s disease [2], has been supported by abundant genetic and pathological evidence. Amyloid precursor protein (APP) is sequentially processed by *β*-secretase (also known as BACE-1) and *γ*-secretase to liberate Aβ. It has been revealed that BACE-1^−/−^ mice are devoid of cerebral Aβ production without any sign of significant dysfunction [3]. In addition, the rescue of memory deficits in BACE-1^−/−^ APP bigenic mice suggested that BACE-1 inhibition would improve Aβ-dependent cognitive impairment in AD patients [4]. Therefore, BACE-1 has been considered to be an attractive target for the therapy of AD with lots of small molecular inhibitors discovered in the past few years. Due to low oral bioavailability, metabolic instability, and poor ability to penetrate brain barrier of peptidomimetic inhibitors, recent attention has been mainly focused on non-peptidomimetic scaffolds, including 1,3,5-trisubstituted aromatics, isophthalamides, acylguanidines, piperazines, macrocycles, amino heterocycles and so on [5,6,7,8,9]. Among compounds with these different scaffolds, aminoheterocyclic derivatives, such as 2-amino-thiazole, 2-aminopyridine, 6-aminoimidazopyrimidine, 2-aminoquinazoline and 2-amino-pyrimidinone, have been of great interest in recent years due to their simple structures and specific binding modes with BACE-1 [10,11,12,13,14,15,16].

**Figure 1:**
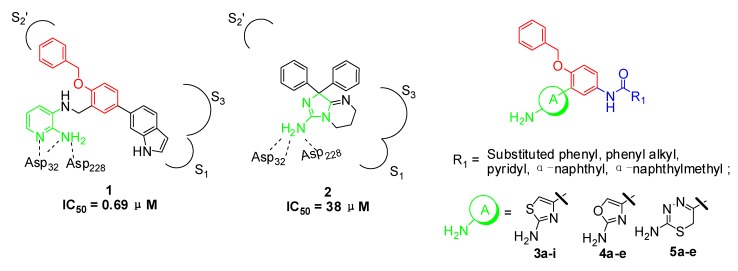
Structures of lead compounds **1**, **2** and designed compounds.

2-Aminopyridine derivative **1** (Figure 1), prepared *via* a fragment based drug design strategy by Congreve and coworkers was reported as a BACE-1 inhibitor with an IC_50_ value of 0.69 μM [13]. The X-ray structure of **1**/BACE-1 complex revealed that **1** occupied two hydrophobic pockets (S_1_ and S_2_’), and the 2-aminopyridine moiety directly interacted with two catalytic aspartic acids (Asp32 and Asp228) *via* two hydrogen bonds (Figure 1). 

Stachel and coworkers revealed that the 2-aminothiazole derivative **2** showed similar binding mode with BACE-1 enzyme in the active site with the amino group interacting directly with Asp32 and Asp228 through a bidentate interaction [14]. Compound **2** exhibited a brain-to-plasma ratio value of 3.9 when it was administered to mice at a 20 mg/kg iv dose (t = 30 min; [brain] = 15 μM). The similar binding features of **1** and **2** with BACE-1 and the desirable brain-barrier penetrating characteristics of compound **2** prompted us to design new amino-heterocyclic derivatives as potent BACE-1 inhibitors by using the following drug design strategies: (1) the 1,2,4-trisubstituted benzene moiety from compound **1** was taken as the skeleton and the 1-benzyloxy moiety was retained to make hydrophobic interactions with S_2_’ binding pocket; (2) 2-aminothiazole, 2-aminooxazole and 2-amino-6*H*-[1,3,4]-thiadiazine moieties were respectively introduced into the 2-position of the benzene based on the bioisosterism principle to form exquisite hydrogen bonds with the two catalytic Asp32 and Asp 228 of BACE-1; (3) various substituted phenyl, pyridyl, phenylalkyl and naphthyl groups were incorporated into the 4-position of the benzene with an amide linkage to fit into the S_1_ or S_3_ binding pocket of BACE-1.

**Figure 2:**
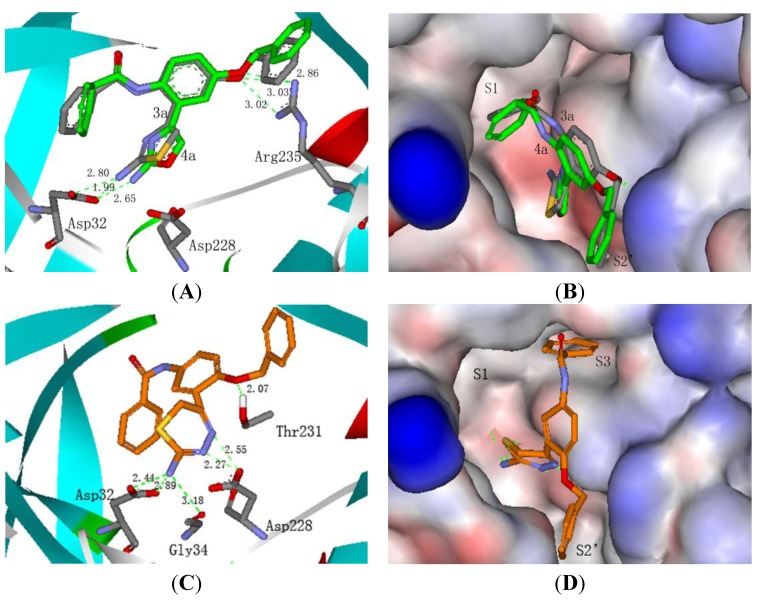
(**A**) Binding mode of **3a** (grey backbone) and **4a** (green backbone) with BACE-1; (**B**) Surface show of **3a** and **4a** bound to BACE-1; (**C**) Binding mode of **5a** with BACE-1; (**D**) Surface show of **5a** bound to BACE-1.

To confirm our hypothesis and predict the binding model of the designed compounds in the active pockets of BACE-1, molecular docking studies of 2-aminothiazole derivative **3a**, 2-aminooxazole derivative **4a** and 2-amino-6*H*-1,3,4-thiadizine derivative **5a** with BACE-1 were performed by using the 2.1/CDOCKER protocol within Discovery Studio package (Figure 2A–D). The crystal structure of ligand/BACE 1 complex (PDB ID: 1W51) was used as the template [15].

As shown in Figure 2A,B, compounds **3a** and **4a** shared similar binding modes with BACE-1. The 1-benzyloxy group was located in the S_2_’ pocket and the benzamide moiety fit within the critical S_1_ pocket. The amino groups of **3a** and **4a** formed hydrogen bonds with Asp32, with distances of 1.99 Å, 2.80 Å and 2.66 Å, respectively. The oxygen atoms of the benzyloxy linkage in **3a** and **4a** formed additional hydrogen bonds with Arg235, with distances of 3.02 Å, 3.03 Å and 2.80 Å, respectively. Interestingly, Figure 2C,D revealed that 2-amino-6*H*-1,3,4-thiadizine derivative **5a** bound to BACE-1 in a different manner. The 4-benzamide moiety extended into the critical S_3_ pocket rather than the S_1_ pocket. The amino group of **5a** not only formed bidentate hydrogen bonds with Asp32 with distances of 2.44 Å and 2.89 Å, but also formed a hydrogen bond with Gly34 with a distance of 3.18 Å. Additionally, the two nitrogen atoms of 6*H*-1,3,4-thiadizine formed bidentate hydrogen bonds with Asp228. Besides, another hydrogen bond was presented between the oxygen atom of the benzyloxy linkage and Thr231. Based on the docking analysis, three series of 3-(2-aminoheterocycle)-4-benzyloxy-phenyl-benzamide derivatives **3a–i**, **4a**–**e** and **5a**–**e** was synthesized and evaluated for their BACE-1 inhibitory activities.

## 2. Results and Discussion

### 2.1. Chemistry

**Scheme 1:**
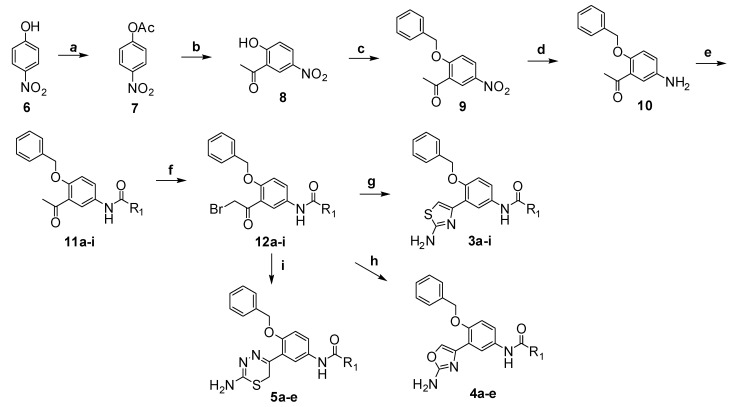
The synthetic route to target compounds **3a**–**i**, **4a**–**e** and **5a**–**e**.

The synthetic route of target compounds **3a**–**i**, **4a**–**e** and **5a**–**e** is outlined in Scheme 1. Treatment of 4-nitrophenol (**6**) with acetic anhydride in aqueous NaOH solution furnished 4-nitrophenyl acetate (**7**), which was converted to 2'-hydroxy-5'-nitro-acetophenone (**8**) through a Fries rearrangement catalyzed by AlCl_3_ in nitrobenzene. Alkylation of **8** with benzyl chloride in the presence of potassium carbonate in ethanol provided 2'-benzyloxy-5'-nitro-acetophenone (**9**), which was reduced using stannous chloride to yield 2'-benzyloxy-5'-amino-acetophenone (**10**). Then, **10** was condensed with different aromatic acyl chlorides to afford amides **11a**–**i**, which were brominated with CuBr_2_ in chloroform to give the α-bromoacetophenone derivatives **12a**–**i**. Finally, **12a**–**i** were condensed with thiourea, urea and aminothiourea in ethanol or DMF, respectively, to get target compounds **3a**–**i**, **4a**–**e** and **5a**–**e**.

*Reagents and conditions*: a. NaOH, (AcO)_2_O; b. AlCl_3_, Ph-NO_2_; c. PhCH_2_Cl, K_2_CO_3_; d. SnCl_2_, EtOH; e. R_1_COCl, Et_3_N; f. CuBr_2_, CHCl_3_, EtOH; g. Thiourea, EtOH, reflux; h. Urea, DMF; i. Aminothiourea, EtOH, reflux.

### 2.2. BACE-1 Inhibitory Activities and *in Vitro* BBB Permeability

#### 2.2.1.BACE-1 Inhibition Activity

The obtained target compounds were tested for their BACE-1 inhibitory activities using a fluorescence resonance energy transfer (FRET) assay, with OM99-2, a potent peptidomimetic inhibitor, as the positive control [16]. Compounds with a BACE-1 inhibition rate higher than 50% at 20 μg/mL were tested for their IC_50_ values. The results are summarized in Table 1.

**Table 1:** The BACE-1 inhibitory activities of **3a**–**e**, **4a**–**e** and **5a**–**e**.

Compd.	Structure	R_1_	% Inhibition at 20 μg/mL (IC_50_ values)
OM99-2			IC_50_ = 0.41 ± 0.12 μM
**3a**	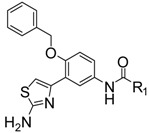	Ph	29.3 ± 1.3
**3b**		4-Cl-Ph	20.4 ± 4.4
**3c**		4-CF_3_-Ph	33.0 ± 1.9
**3d**		4-OMe-Ph	29.5 ± 3.5
**3e**		α-Naphth	55.3 ± 5.7 (IC_50_ > 20 μM)
**3f**		3-Py	36.8 ± 6.9
**3g**		–CH_2_Ph	8.9 ± 7.0
**3h**		–CH_2_CH_2_Ph	3.1 ± 2.5
**3i**		–CH_2_-α-Naphth	11.3 ± 0.7
**4a**	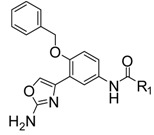	Ph	38.8 ± 0.3
**4b**		4-Cl-Ph	49.9 ± 0.2
**4c**		4-CF_3_-Ph	47.5 ± 9.9
**4d**		4-OMe-Ph	34.4 ± 5.7
**4e**		α-Naphth	42.6 ± 3.2
**5a**	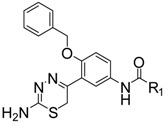	Ph	64.2 ± 0.5 (IC_50_ = 16.7 ± 4.4 μM)
**5b**		4-Cl-Ph	76.0 ± 2.5 (IC_50_ > 20 μM)
**5c**		4-CF_3_-Ph	46.9 ± 7.5
**5d**		4-OMe-Ph	84.9 ± 16.7 (IC_50_ > 20 μM)
**5e**		α-Naphth	60.0 ± 3.1 (IC_50_ = 9.9 ± 1.7 μM)

As shown in Table 1, most of the tested compounds demonstrated moderate to good BACE-1 inhibition at 20 μg/mL, 13 compounds exhibited more than 30% inhibition and five compounds showed more than 50% inhibition. Preliminary structure-activity relationships could be concluded as follows:

(1) The variation of the heterocycle moiety affected the BACE-1 inhibitory activities significantly. 2-Amino-6*H*-1,3,4-thiadizine derivatives were more potent than 2-aminothioazole and 2-amino-oxazole derivatives. Four of the 2-amino-6*H*-1,3,4-thiadizine derivatives (compounds **5a**, **5b**, **5d** and **5e**) showed more than 60% inhibition against BACE-1 at 20 μg/mL (64.2, 76.0, 84.9 and 60.0%, respectively). Among the synthesized compounds, **5a** and **5e** are the two most potent BACE-1 inhibitors, with IC_50_ values of 16.7 and 9.9 μM, respectively. The 2-aminooxazole derivatives **4a**–**e** demonstrated similar BACE-1 inhibitory activities as the 2-aminothioazoles **3a**–**e**, with inhibition rates ranging from 34.4% to 49.9% in comparison with 20.4% to 55.3% at 20 μg/mL. The previous docking study of compounds **3a**, **4a** and **5a** with BACE-1 reveals that the enhanced potency of 2-amino-thiadiazine derivatives **5a**–**e** may be attributed to their different binding modes with BACE-1 than the 2-aminothiazole and 2-aminooxazole derivatives, with the amino group forming two additional hydrogen bonds with Gly34 and Asp228, and the benzamide moiety fitting into the S_3_ pocket instead of the S_1_ pocket (Figure 2A,B *versus* Figure 2C,D).

(2) A glimpse of different substituents at the R_1_-position implied that α-naphthyl group introduction was favorable for BACE-1 inhibition. For example, **3e**, **4e** and **5e** exhibited 55.3%, 42.6% and 60.0% of BACE-1 inhibition at 20 μg/mL, respectively, and **5e** showed the lowest IC_50_ value (9.9 μM) among all the compounds. This implied that a bulky naphthyl ring was better accommodated into the critical S_1_ or S_3_ binding pockets and an electron-rich ring had a better hydrophobic interaction with amino acids. The existence of chloro, methoxy or trifluoromethyl phenyl ring substituents had a limited effect on BACE-1 inhibitory activities, for example, 2-aminothiazole derivatives **3a**–**d** showed similar BACE-1 inhibition rates, ranging from 20.4% to 33.0%.

(3) The introduction of CH_2_ or (CH_2_)_2_ linkages between the amide and aromatic moiety resulted in a significant decrease in BACE-1 inhibition. For example, benzamide derivative **3a** showed 29.3% BACE-1 inhibition at 20 μg/mL, which was about three fold more potent than that of phenylacetamide derivative **3g** (8.9%), and about ten fold more potent than that of phenylpropanamide derivative **3h** (3.1% inhibition). α-Naphthyl amide derivative **3e** also showed much more potent BACE-1 inhibitory activity (55.3%) than that of the α-naphthylacetamide derivative **3i (**11.3%).

#### 2.2.2.*In Vitro,* Blood-Brain Barrier Permeability

Blood-brain barrier (BBB) permeation is critical for any AD therapeutic drug. Many previously synthesized potent BACE-1 inhibitors displayed poor brain barrier penetration, which restricted their further development. For example, the highly potent BACE-1 inhibitor GSK188909 (IC_50_ = 5.0 nM) showed poor blood-brain barrier permeability, and it need to be combined with Pgp inhibitor GF120918 to exert its Aβ reducing activity in the brain of mice [17]. In order to investigate the BBB permeability of the newly synthesized aminoheterocyclic derivatives, the most potent compound **5e** was picked out to evaluate its transport efficient (P_app_ values) in Madin-Darby canine kidney cell line (MDCK) and Madin-Darby canine kidney-multidrug resistance 1 (MDCK-MDR1) monolayer cells (*in vitro* cell culture model of BBB). The results are summarized in Table 2.

**Table 2:** The transport efficient (Papp values) of **5e** across MDCK and MDCK-MDR1 cells.

MDCK			MDCK-MDR1			
Papp (×10^−6^ cm/s)		Efflux ratio	Papp (×10^−6^ cm/s)		Efflux ratio	Net efflux ratio
A-B	B-A		A-B	B-A		
28.20 ± 6.45	27.66 ± 2.87	0.98	31.78 ± 1.85	22.23 ± 1.24	0.70	0.71

Concentration of **5e** was 55.6 μM, transport efficient (P_app_value) are presented as the mean ± SD; n = 3.

As shown in Table 2, compound **5e** exhibited high apparent permeability coefficients *P*_app_(A-B) and *P*_app_(B-A) in the MDCK cell model, with values of 28.20 × 10^−6^ cm/s and 27.66 × 10^−6^ cm/s. Similarly, **5e** also exhibited *P*_app_(A-B) and *P*_app_(B-A) values of 31.78 × 10^−6^ cm/s and 22.23 × 10^−6^ cm/s in the MDCK-MDR1 cell model, respectively. It has been reported that compounds with *P*_app_(A-B) values > 3 × 10^−6^ cm/s in the MDCK-MDR1 model would have high brain uptake potential [18]. These results suggested that compound **5e** had a good penetration ability through the blood-brain barrier. The efflux ratios of **5e** in the MDCK and MDCK-MDR1 models were 0.98 and 0.70, respectively. The net efflux ratio of **5e** was 0.71, less than FDA’s recommendation of 2 for P-gp substrate, which indicated that compound **5e** was not a P-gp substrate [19].

## 3. Experimental

### 3.1. General

All reagents and solvents used were analytical grade and purchased from common commercial suppliers. Melting points were determined with a B-540 Buchi melting-point apparatus and are uncorrected. ^1^H-NMR was performed on a Brüker Advance DMX 400 MHz spectrometer with TMS as internal standard. Proton chemical shifts were expressed in parts per million (ppm) and coupling constants in Hz. HRMS spectra were measured with an Agilent 6224 TOF LC/MS. Mass spectra (ESI-MS, positive) were recorded on a Finnigan LCQ DecaXP ion trap mass spectrometry. Molecular docking studies were performed using Discovery Studio 2.1.

### 3.2. Chemistry

*4-Nitrophenyl acetate* (**7**). To a warmed (90–95 °C) mixture of 4-nitrophenol (**6**, 2.78 g, 0.02 mol) in aqueous NaOH solution (20 mL, 1.5 mol/L) was added Ac_2_O (2.83 mL, 0.03 mol). The mixture was stirred and cooled to room temperature. The formed precipitate was collected by suction filtration, washed with water and dried *in vacuo* to afford **7** as a pale yellow solid (3.52 g, 97.2%), m.p. 78–80 °C (lit. 77–79 °C) [20].

*2'-Hydroxy-5'-nitroacetophenone* (**8**). To a stirred solution of AlCl_3_ (1.6 g, 0.012 mmol) in dry nitrobenzene (15 mL) was added 4-nitrophenyl acetate (**7**, 2.0 g, 0.011 mol), and the mixture was heated at 140 °C for 6 h. Upon cooling, the mixture was poured into a beaker with crushed ice (15 g) and conc. hydrochloric acid (6.0 mL). The organic layer was separated and washed with 10% NaOH (10 mL × 2). The obtained aqueous layers were acidified to pH = 5 with diluted hydrochloric acid and extracted with ethyl acetate. The combined organic layer was evaporated under vacuum and the residue was purified by silica gel chromatography eluting with PE-EtOAc (15:1, v/v) to provide **8** as a light pink solid, 0.88 g, yield 43.5%, m.p. 101–103 °C (lit. 101–102 °C) [21].

*2'-Benzyloxy-5'-nitroacetophenone* (**9**). A mixture of 2'-hydroxy-5'-nitroacetophenone (**8**, 0.72 g, 4.0 mmol), K_2_CO_3_ (0.58 g, 4.2 mmol), benzyl chloride (0.6 g, 4.7 mmol), a catalytic amount of KI and TEBA in CH_3_CN (12.0 mL) and H_2_O (1.6 mL) was stirred and refluxed for 2 h. The solvent was removed *in vacuo* and the residue partitioned between ethyl acetate (30 mL) and water (20 mL). The organic layer was separated and washed with brine, dried over Na_2_SO_4_ and evaporated to afford crude **9**, which was recrystallized with ethanol to yield 1.01 g of a yellow solid, yield 92.4%, m.p. 112–114 °C. ^1^H-NMR (CDCl_3_): δ 2.61 (s, 3H, CH_3_), 5.28 (s, 2H, CH_2_), 7.12 (d, 1H, *J* = 7.2 Hz, Ar-H), 7.40 (m, 1H, Ar-H), 7.43 (m, 4H, Ar-H), 8.32 (dd, 1H, *J_1_* = 7.2 Hz, *J_2_* = 3.0 Hz, Ar-H), 8.62 (d, 1H, *J* = 3.0 Hz, Ar-H). ESI-MS: *m/z* = 272.3 [M+H]^+^.

*2-Benzyloxy-5-aminoacetophenone* (**10**). To a mixture of 2-benzyloxy-5-nitroacetophenone (**9**, 0.81 g, 3.0 mmol) in EtOH (15 mL) was added SnCl_2_·2H_2_O (3.39 g, 15.0 mmol) in portions, and the resulting mixture was warmed to 45 °C for 3 h. The solvent was removed *in vacuo* and the residue was partitioned between ethyl acetate (20 mL) and 10% NaOH solution (15 mL). The organic layer was separated, washed with saline, dried over anhydrous sodium sulfate, and evaporated *in vacuo* to afford **10** as a pale yellow oil (0.63 g, 87%). ^1^H-NMR (CDCl_3_): δ 2.57 (s, 3H, CH_3_), 3.49 (bs, 2H, NH_2_), 5.07 (s, 2H, CH_2_), 6.78 (dd, 1H, *J*_1_ = 7.2 Hz, *J*_2_ =3.0 Hz, Ar-H), 6.85 (d, 1H, *J* = 7.2 Hz, Ar-H), 7.07 (d, 1H, *J* = 3.0 Hz, Ar-H), 7.33–7.36 (m, 1H, Ar-H), 7.39–7.42 (m, 4H, Ar-H). ESI-MS: *m/z* = 242.2 [M+H]^+^.

#### 3.2.1. General Procedure for the Synthesis of **11a–i**

To a mixture of 2-benzyloxy-5-aminoacetophenone (**10**, 0.72 g, 3.0 mmol) and Et_3_N (0.87 mL, 6.0 mmol) in CH_2_Cl_2_ (10 mL), a solution of aromatic acyl chlorides (4.5 mmol) in CH_2_Cl_2_ (10 mL) was added in dropwise within 30 min. The mixture was stirred for 6–8 h at room temperature. Then, saturated NaHCO_3_ solution (20 mL) was added and organic layer was separated, washed with brine and dried over anhydrous sodium sulphate. The solvent was removed under vacuum and the residue was purified by silica gel chromatography eluting with PE-EtOAc (5:1) to give **11a**–**i**.

*N-(3-Acetyl-4-(benzyloxy)phenyl)benzamide* (**11a**). White solid, yield 83%, m.p.: 139–142 °C. ^1^H-NMR (CDCl_3_): δ 2.61 (s, 3H, CH_3_), 5.17 (s, 2H, CH_2_), 7.06 (d, *J* = 7.2 Hz, 1H, Ar-H), 7.31–7.34 (m, 1H, Ar-H), 7.40–7.43 (m, 3H, Ar-H), 7.44–7.47 (m, 3H, Ar-H), 7.52–7.54 (m, 1H, Ar-H), 7.71 (d, 1H, *J* = 7.2 Hz, Ar-H), 7.86 (d, 2H, *J* = 7.6 Hz, Ar-H), 8.09 (brs, 1H, NH), 8.17 (dd, 1H, *J*_1_ = 8.0 Hz, *J*_2_ = 3.0 Hz, Ar-H). ESI-MS: *m/z* = 346.4 [M+H]^+^.

*N-(3-Acetyl-4-(benzyloxy)phenyl)-4-chlorobenzamide* (**11b**). White solid, yield 80%, m.p.: 190–192 °C. ^1^H-NMR (CDCl_3_): δ 2.56 (s, 3H, CH_3_), 5.20 (s, 2H, CH_2_), 6.96 (d, *J* = 7.2 Hz, 1H, Ar-H), 7.37 (t, 1H, *J* = 5.6 Hz, Ar-H), 7.38–7.39 (m, 2H, Ar-H), 7.44–7.47 (m, 2H, Ar-H), 7.54 (d, 2H, *J* = 6.8 Hz, Ar-H), 7.74 (d, 1H, *J* = 7.2 Hz, Ar-H), 7.91 (d, 2H, *J* = 6.8 Hz, Ar-H), 8.17 (dd, 1H, *J* = 7.2 Hz, *J*_2_ = 3.0 Hz, Ar-H), 8.25 (brs, 1H, NH). ESI-MS: *m/z* = 380.8 [M+H]^+^.

*N-(3-Acetyl-4-(benzyloxy)phenyl)-4-trifluoromethylbenzamide* (**11c**). White solid, yield 88%, m.p.: 130–134 °C. ^1^H-NMR (CDCl_3_): δ 2.58 (s, 3H, CH_3_), 5.24 (s, 2H, CH_2_), 7.08 (d, 1H, *J* = 7.2 Hz, Ar-H), 7.39–7.42 (m, 2H, Ar-H), 7.47–7.50 (m, 3H, Ar-H), 7.78 (m, 3H, Ar-H), 7.94 (brs, 1H, NH), 8.02 (d, 2H, *J* = 6.4 Hz, Ar-H), 8.21 (d, 1H, *J* = 7.2 Hz, Ar-H). ESI-MS: *m/z* = 414.5 [M+H]^+^.

*N-(3-Acetyl-4-(benzyloxy)phenyl)-4-methoxybenzamide* (**11d**). Yellow solid, yield 80%, m.p.: 150–152 °C. ^1^H-NMR (CDCl_3_): δ 2.56 (s, 3H, CH_3_), 3.68 (s, 3H, OCH_3_), 5.21 (s, 2H, CH_2_), 6.91 (d, 1H, *J* = 7.2 Hz, Ar-H), 7.03 (d, 2H, *J* = 7.2 Hz, Ar-H), 7.36–7.43 (m, 3H, Ar-H), 7.51 (d, 2H, *J* = 5.6 Hz, Ar-H), 7.75 (d, 1H,*J* = 7.2 Hz, Ar-H), 7.94 (d, 2H, *J* = 7.2 Hz, Ar-H), 8.12 (d, 1H, *J* = 3.0 Hz, Ar-H), 8.23 (brs, 1H, NH). ESI-MS: *m/z* = 376.4 [M+H]^+^.

*N-(3-Acetyl-4-(benzyloxy)phenyl)-1-naphthamide* (**11e**). White solid, yield 85%, m.p.: 135–136 °C. ^1^H-NMR (CDCl_3_): δ 2.56 (s, 3H, CH_3_), 5.18 (s, 2H, CH_2_), 7.08 (d, 1H, *J* = 7.2 Hz, Ar-H), 7.35 (m, 2H, Ar-H), 7.40–744 (m, 3H, Ar-H), 7.49–7.55 (m, 3H, Ar-H), 7.68–7.71 (m, 2H, Ar-H), 7.85–7.88 (m, 2H, Ar-H), 7.94 (d, 1H, *J* = 6.8 Hz, Ar-H), 8.19 (d, 1H, *J* = 6.4 Hz, Ar-H), 8.34 (d, 1H, *J* = 6.4 Hz, Ar-H). ESI-MS: *m/z* = 396.5 [M+H]^+^.

*N-(3-Acetyl-4-benzyloxyphenyl)nicotinamide* (**11f**). Pale yellow solid, yield 73%, m.p.: 160–162 °C. ^1^H-NMR (CDCl_3_): δ 2.61 (s, 3H, CH_3_), 5.19 (s, 2H, CH_2_), 7.09 (d, 1H, *J* = 7.2 Hz, Ar-H), 7.38–7.40 (m, 1H, Ar-H), 7.46–7.49 (m, 5H, Ar-H), 7.79 (d, 1H, *J* = 7.2 Hz, Ar-H), 8.18 (d, 1H, *J* = 3.0 Hz, Py-H), 8.24 (d, 1H, *J* = 1.5 Hz, Py-H), 8.38 (brs, 1H, NH), 8.76 (d, 1H, *J* = 2.0 Hz, Py-H), 9.14 (s, 1H, Py-H). ESI-MS: *m/z* = 347.4 [M+H]^+^.

*N-(3-Acetyl-4-(benzyloxy)phenyl)-2-phenylacetamide* (**11g**). White solid, yield 62%, mp: 156–157 °C. ^1^H-NMR (CDCl_3_): δ 2.57 (s, 3H, CH_3_), 3.71 (s, 2H, CH_3_), 5.16 (s, 2H, CH_2_), 7.03 (d, 1H, *J* = 3.6 Hz, Ar-H), 7.08 (s, 1H, Ar-H), 7.35 (d, 2H, *J*= 6.0 Hz, Ar-H), 7.34–7.39 (m, 2H, Ar-H), 7.41 (d, 3H, *J* = 6.4 Hz, Ar-H), 7.43 (d, 3H, *J* = 7.2 Hz, Ar-H), 7.46 (m, 1H, Ar-H), 8.06 (dd, 1H, *J_1_* = 7.2 Hz, *J_2_* = 2.4 Hz, Ar-H). ESI-MS: *m/z* = 360.4 [M+H]^+^.

*N-(3-Acetyl-4-benzyloxyphenyl)-3-phenylpropionamide* (**11h**). White solid, yield 57%, m.p.: 133–135 °C. ^1^H-NMR (CDCl_3_): δ 2.55 (s, 3H, *J* = 8.0Hz, CH_3_), 2.67 (t, 2H,*J* = 6.4 Hz, CH_2_), 3.06 (t, 2H, *J* = 6.4 Hz, CH_2_), 5.17 (s, 2H, CH_2_), 7.03 (d, 1H, *J* = 7.2 Hz, Ar-H), 7.26–7.29 (m, 4H, Ar-H), 7.33-–7.36 (m, 3H, Ar-H), 7.41–7.45 (m, 4H, Ar-H), 7.49 (d, 1H, *J* = 7.5 Hz, Ar-H), 7.81 (d, 1H, *J* = 2.5 Hz, Ar-H). ESI-MS: *m/z* = 374.5 [M+H]^+^.

*N-(3-Acetyl-4-(benzyloxy)phenyl)-2-(naphthalen-1-yl)acetamide* (**11i**). White solid, yield 75%, m.p.: 121–122 °C. ^1^H-NMR (CDCl_3_): δ 2.56 (s, 3H, CH_3_), 3.91 (s, 2H, CH_2_), 5.21 (s, 2H, CH_2_), 6.88 (d, 1H, *J* = 7.2 Hz, Ar-H), 7.15 (d, 1H, *J* = 7.2 Hz, Ar-H), 7.18–7.20 (m, 1H, Ar-H), 7.28–7.33 (m, 2H, Ar-H), 7.43–7.45 (m, 3H, Ar-H), 7.46–7.48 (m, 3H, Ar-H), 7.52 (d, 1H, *J* = 7.2 Hz, Ar-H), 7.78 (d, 1H, *J* = 6.4 Hz, Ar-H), 7.94 (d, 1H, *J* = 6.0 Hz, Ar-H), 8.19 (d, 1H, *J* = 6.4 Hz, Ar-H), 8.35 (d, 1H, *J* = 7.2 Hz, Ar-H). ESI-MS: *m/z* = 410.4 [M+H]^+^.

#### 3.2.2. General Procedure for the Synthesis of **12a–i**

To a refluxed solution of *N*-(3-acetyl-4-(benzyloxy)phenyl)amide derivatives **11a**–**i** (2.0 mmol) in 1:1 EtOH–CH_3_Cl (15 mL) was added CuBr_2_ (4.0 mmol) in three portions within 2 h. The mixture was cooled to room temperature and filtered. The filtrate was concentrated to dryness and the residue was extracted with EtOAc twice. The organic layer was combined, washed with brine, dried with Na_2_SO_4_, evaporated *in vacuo* to dryness. The residue was purified with silica gel chromatography eluting with PE–EtOAc (5:1) to afford **12a**–**i**.

*N-(4-(Benzyloxy)-3-(2-bromoacetyl)phenyl)benzamide* (**12a**). Yellow solid, yield 79%, m.p.: 183–186 °C. ^1^H-NMR (CDCl_3_): δ 4.53 (s, 2H, CH_2_), 5.19 (s, 2H, CH_2_), 7.08 (d, 1H, *J* = 7.2 Hz, Ar-H), 7.36–7.39 (m, 1H, Ar-H), 7.40–7.43 (m, 3H, Ar-H), 7.45–7.48 (m, 3H, Ar-H), 7.51–7.53 (m, 1H, Ar-H), 7.72 (d, 1H, *J* = 7.2 Hz, Ar-H), 7.86 (d, 2H, *J* = 7.6 Hz, Ar-H), 7.97 (s, 1H, NH), 8.20 (dd, 1H, *J_1_* = 7.6 Hz, *J_2_* = 2.0 Hz, Ar-H). ESI-MS: *m/z* = 425.4[M+H]^+^.

*N-(4-(Benzyloxy)-3-(2-bromoacetyl)phenyl)-4-chlorobenzamide* (**12b**). Yellow solid, yield 82%, m.p.: 198–199 °C. ^1^H-NMR (CDCl_3_): δ 4.56 (s, 2H, CH_2_), 5.21 (s, 2H, CH_2_), 7.06 (d, *J* = 7.2 Hz, 1H, Ar-H), 7.40–7.43 (m, 3H, Ar-H), 7.48 (d, 2H, *J* = 5.6 Hz, Ar-H), 7.52 (d, 2H, *J* = 6.8 Hz, Ar-H), 7.72 (d, 1H, *J* = 7.2 Hz, Ar-H), 7.86 (d, 2H, *J* = 6.8 Hz, Ar-H), 8.13 (s, 1H, NH), 8.24 (dd, 1H, *J_1_* = 7.2 Hz, *J_2_* = 3.0 Hz, Ar-H). ESI-MS: *m/z* = 459.9 [M+H]^+^.

*N-(4-(Benzyloxy)-3-(2-bromoacetyl)phenyl)-4-(trifluoromethyl)benzamide* (**12c**).Yellow solid, yield 80%, m.p.:157–159 °C. ^1^H-NMR (CDCl_3_): δ 4.54 (s, 2H, CH_2_), 5.22 (s, 2H, CH_2_), 7.13 (d, 1H, *J* = 7.2 Hz, Ar-H), 7.40–7.50 (m, 5H, Ar-H), 7.76–7.78 (m, 3H, Ar-H), 7.92 (s, 1H, NH), 8.00 (d, 2H, *J* = 6.4 Hz, Ar-H), 8.19 (dd, 1H, *J* = 7.2 Hz, *J_2_* = 2.6 Hz, Ar-H). ESI-MS: *m/z* = 493.3 [M+H]^+^.

*N-(4-(Benzyloxy)-3-(2-bromoacetyl)phenyl)-4-methoxybenzamide* (**12d**).Yellow solid, yield 75%, m.p.: 158–159 °C. ^1^H-NMR (CDCl_3_): δ 3.87 (s, 3H, CH_3_), 4.55 (s, 2H, CH_2_), 5.22 (s, 2H, CH_2_), 7.02 (d, 1H, *J* = 7.2 Hz, Ar-H), 7.12 (d, 2H, *J* = 7.2 Hz, Ar-H), 7.40–7.42 (m, 1H, Ar-H), 7.46–7.49 (m, 4H, Ar-H), 7.92 (s, 1H, Ar-H), 7.94 (d, 2H, *J* = 7.2 Hz, Ar-H), 8.07 (s, 1H, NH), 8.15 (d, 1H, *J* = 3.0 Hz, Ar-H). ESI-MS: *m/z* = 455.6 [M+H]^+^.

*N-(4-(Benzyloxy)-3-(2-bromoacetyl)phenyl)-1-naphthamide* (**12e**).Yellow solid, yield 80%, m.p.: 155–156 °C. ^1^H-NMR (δ, CDCl_3_): 4.55 (s, 2H, CH_2_), 5.18 (s, 2H, CH_2_), 7.07 (d, 1H, *J* = 7.2 Hz, Ar-H), 7.35 (d, 2H, *J* = 3.6 Hz, Ar-H), 7.41–7.45 (m, 3H, Ar-H), 7.48–7.56 (m, 3H, Ar-H), 7.70–.73 (m, 2H, Ar-H), 7.93–7.95 (m, 2H, Ar-H, NH), 7.98 (d, 1H, *J* = 6.4 Hz, Ar-H), 8.21 (d, 1H, *J* = 5.6 Hz, Ar-H), 8.35 (d, 1H, *J* = 6.4 Hz, Ar-H). ESI-MS: *m/z* = 476.1 [M+H]^+^.

*N-(4-(Benzyloxy)-3-(2-bromoacetyl)phenyl)nicotinamide* (**12f**). Yellow solid, yield 51%, m.p.: 148–149 °C. ^1^H-NMR (δ, CDCl_3_): 4.55 (s, 2H, CHB_2B_), 5.21 (s, 2H, CHB_2B_), 7.11 (d, 1H, *J* = 7.2 Hz, Ar-H), 7.36–7.48 (m, 6H, Ar-H), 7.71 (d, 1H, *J* = 7.2 Hz, Ar-H), 8.13 (d, 1H, *J* = 3.0 Hz, Ar-H), 8.28 (d, 1H, *J* = 1.5 Hz, Ar-H), 8.36 (s, 1H, Ar-H), 8.71 (d, 1H, *J* = 2.0 Hz, Ar-H), 8.99 (s, 1H, NH). ESI-MS: *m/z* = 426.4 [M+H]^+^.

*N-(4-(Benzyloxy)-3-(2-bromoacetyl)phenyl)-2-phenylacetamide* (**12g**). Yellow solid, yield 79%, m.p.: 178–179 °C. ^1^H-NMR (CDCl_3_): δ 3.73 (s, 2H, CH_3_), 4.48 (s, 2H, CH_2_), 5.16 (s, 2H, CH_2_), 7.01 (d, 1H, *J* = 3.6 Hz, Ar-H), 7.06 (s, 1H, Ar-H), 7.33–7.36 (m, 4H, Ar-H), 7.39–7.42 (m, 7H, Ar-H), 8.03 (dd, 1H, *J_1_* = 7.2 Hz, *J_2_* = 2.4 Hz, Ar-H). ESI-MS: *m/z* = 439.4 [M+H]^+^.

*N-(4-(Benzyloxy)-3-(2-bromoacetyl)phenyl)-3-phenylpropanamide* (**12h**). Yellow solid, yield 72%, m.p.: 153–155 °C. ^1^H-NMR (δ, CDCl_3_): 2.67 (t, 2H,*J* = 6.4 Hz, CHB_2B_), 3.06 (t, 2H, *J* = 6.4 Hz, CHB_2B_), 4.51 (s, 2H, CHB_2B_), 5.16 (s, 2H, CHB_2B_), 7.02 (d, 1H, *J* = 7.2 Hz, Ar-H), 7.24–7.27 (m, 4H, Ar-H), 7.32–7.38 (m, 4H, Ar-H), 7.42–7.46 (m, 3H, Ar-H), 7.48 (d, 1H, *J* = 7.2 Hz, Ar-H), 7.99 (d, 1H, *J* = 6.8 Hz, Ar-H). ESI-MS: *m/z* = 453.3 [M+H]^+^.

*N-(4-(Benzyloxy)-3-(2-bromoacetyl)phenyl)-2-(naphthalen-1-yl)acetamide* (**12i**). Yellow solid, yield 70%, m.p.:144–148 °C. ^1^H-NMR (CDCl_3_): δ 3.93 (s, 2H, CH_2_), 4.56 (s, 2H, CH_2_), 5.22 (s, 2H, CH_2_), 6.89 (d, 1H, *J* = 7.2 Hz, Ar-H), 7.16 (d, 1H, *J* = 7.2 Hz, Ar-H), 7.18–7.20 (m, 1H, Ar-H), 7.39–7.42 (m, 2H, Ar-H), 7.42–7.44 (m, 3H, Ar-H), 7.46–7.49 (m, 3H, Ar-H), 7.54 (d, 1H, *J* = 7.2 Hz, Ar-H), 7.77 (d, 1H, *J* = 6.4 Hz, Ar-H), 7.94 (d, 1H, *J* = 6.0 Hz, Ar-H), 8.19 (d, 1H, *J* = 6.4 Hz, Ar-H), 8.38 (d, 1H, *J* = 7.2 Hz, Ar-H). ESI-MS: *m/z* = 489.5 [M+H]^+^.

#### 3.2.3. General Procedure for the Synthesis of *N*-(3-(2-Aminothiazol-4-yl)-4-(benzyloxy)phenyl)-amides **3a–i**

The mixture of bromoacetophenone derivative **12a**–**i** (0.2 mmol) and thiourea (0.22 mmol) in EtOH (10 mL) was refluxed for 6–8 h until the substrate **12a**–**i** had disappeared. After cooling to room temperature, the mixture was filtered and the filtrate was concentrated to dryness. The residue was purified by silica gel chromatography eluting with PE-EtOAc-Et_3_N (100:50:1) to afford white to pale yellow solids.

*N-(3-(2-Aminothiazol-4-yl)-4-(benzyloxy)phenyl)benzamide* (**3a**). White solid, yield 93%, m.p.: 239–241 °C. IR (KBr) 3372, 3200, 3073, 1640, 1526 cm^−1^. ^1^H-NMR (DMSO-*d*_6_): δ 5.24 (s, 2H, CH_2_), 7.08 (s, 1H, Ar-H), 7.24 (d, 1H, *J* = 8.8 Hz, Ar-H), 7.31–7.33 (m, 1H, Ar-H), 7.37–7.39 (m, 2H, Ar-H), 7.44–7.46 (m, 2H, Ar-H), 7.51–7.57 (m, 3H, Ar-H), 7.67 (dd, 1H, *J_1_* = 7.2 Hz, *J_2_* = 2.4 Hz, Ar-H), 7.95 (d, 2H, *J* = 7.2 Hz, Ar-H), 8.10 (s, 1H, Ar-H), 8.35 (brs, 2H, NH_2_), 10.31 (brs, 1H, NH). HRMS (ESI) calculated for C_23_H_20_N_3_O_2_S [M+H]^+^: 402.1271, found: 402.1266.

*N-(3-(2-Aminothiazol-4-yl)-4-(benzyloxy)phenyl)-4-chlorobenzamide* (**3b**). White solid, yield 91%, m.p.: 246–247 °C. IR (KBr) 3352, 3195, 3032, 1644, 1518 cm^−1^. ^1^H-NMR (DMSO-*d*_6_): δ 5.29 (s, 2H, CH_2_), 7.13 (s, 1H, Ar-H), 7.31 (d, 1H, *J* = 7.2 Hz, Ar-H), 7.39–7.42 (m, 1H, Ar-H), 7.45–7.47 (m, 2H, Ar-H), 7.51 (d, 2H, *J* = 6.0 Hz, Ar-H), 7.66 (d, 2H, *J* = 7.2 Hz, Ar-H), 7.73–7.75 (m, 1H, Ar-H), 8.05 (d, 2H, *J* = 6.8 Hz, Ar-H), 8.13 (s, 1H, Ar-H), 8.38 (brs, 2H, NH_2_), 10.39 (s, 1H, CONH). ^13^C-NMR (DMSO-*d*_6_): δ 169.53, 164.63, 152.18, 137.16, 136.92, 133.78, 132.77, 130.05, 128.99, 128.38, 128.01, 123.61, 122.33, 114.20, 106.01, 70.56. HRMS (ESI) calculated for C_23_H_19_ClN_3_O_2_S [M+H]^+^: 436.0881, found: 436.0885.

*N-(3-(2-Aminothiazol-4-yl)-4-(benzyloxy)phenyl)-4-(trifluoromethyl)benzamide* (**3c**). White solid, yield 85%, m.p.: 241–243 °C. IR (KBr) 3384, 3271, 3040, 1645, 1518 cm^−1^. ^1^H-NMR (DMSO-*d*_6_): δ 5.24 (s, 2H, CH_2_), 7.10 (s, 1H, Ar-H), 7.23–7.25 (m, 1H, Ar-H), 7.35–7.37 (m, 1H, Ar-H), 7.41–7.44 (m, 2H, Ar-H), 7.48 (d, 2H, *J* = 6.4 Hz, Ar-H), 7.66–7.68 (m, 1H, Ar-H), 7.91 (d, 2H, *J* = 6.8 Hz, Ar-H), 8.16 (d, 2H, *J* = 6.4 Hz, Ar-H), 8.23 (s, 1H, Ar-H), 10.45 (s, 1H, CONH). ^13^C-NMR (DMSO-*d*_6_): δ 172.70, 170.37, 169.85, 164.58, 152.24, 138.86, 137.53, 137.21, 137.11, 132.67, 129.05, 128.98, 128.82, 128.37, 128.00, 127.80, 127.72, 127.62, 125.89, 122.29, 114.32, 105.95, 70.55. HRMS (ESI) calculated for C_24_H_19_F_3_N_3_O_2_S [M+H]^+^: 470.1145, found: 470.1141.

*N-(3-(2-Aminothiazol-4-yl)-4-(benzyloxy)phenyl)-4-methoxybenzamide* (**3d**). Yellow solid, yield 88%, m.p.: 202–203 °C. IR (KBr) 3342, 3195, 3035, 1645, 1503 cm^−1^. ^1^H-NMR (DMSO): δ 3.83 (s, 3H, CH_3_), 5.23 (s, 2H, CH_2_), 6.90 (s, 2H, NH_2_), 7.04 (d, 2H, *J* = 7.2 Hz, Ar-H), 7.10 (s, 1H, Ar-H), 7.13 (d, 1H, *J* = 7.2 Hz, Ar-H), 7.33–7.35 (m, 1H, Ar-H), 7.40–7.42 (m, 2H, Ar-H), 7.51 (d, 2H, *J* = 5.6 Hz, Ar-H), 7.58 (dd, 1H, *J_1_* = 7.2 Hz, *J_2_* = 2.4 Hz, Ar-H), 7.98 (d, 2H, *J* = 7.2 Hz, Ar-H), 8.38 (d, 1H, *J* = 2.4 Hz, Ar-H), 10.01 (s, 1H, CONH). HRMS (ESI) calculated for C_24_H_22_N_3_O_3_S [M+H]^+^: 432.1376, found: 432.1369.

*N-(3-(2-Aminothiazol-4-yl)-4-(benzyloxy)phenyl)-1-naphthamide* (**3e**). Brown solid, yield 88%, m.p.: 198–200 °C. IR (KBr) 3300, 3193, 3031, 1632, 1505 cm^−1^. ^1^H-NMR (DMSO-*d_6_*): δ 5.25 (s, 2H, CH_2_), 6.92 (s, 2H, NH_2_), 7.12 (s, 1H, Ar-H), 7.17 (d, 1H, *J* = 6.8 Hz, Ar-H), 7.36–7.39 (m, 1H, Ar-H), 7.43–7.45 (m, 2H, Ar-H), 7.51 (d, 2H, *J* = 6.0 Hz, Ar-H), 7.56–7.61 (m, 4H, Ar-H), 7.74 (d, 1H, *J* = 5.6 Hz, Ar-H), 8.00–8.02 (m, 1H, Ar-H), 8.06 (d, 1H, *J* = 6.4 Hz, Ar-H), 8.21–8.23 (m, 1H, Ar-H), 8.49 (d, 1H, *J* = 1.6 Hz, Ar-H), 10.42 (s, 1H, CONH). HRMS (ESI) calculated for C_27_H_22_N_3_O_2_S [M+H]^+^: 452.1427, found: 452.1428.

*N-(3-(2-Aminothiazol-4-yl)-4-(benzyloxy)phenyl)nicotinamide* (**3f**). Yellow solid, yield 79%, m.p.: 212–213 °C. IR (KBr) 3367, 3203, 3029, 1673, 1523 cm^−1^. ^1^H-NMR (DMSO-*d_6_*): δ 5.28 (s, 2H, CH_2_), 6.95 (s, 2H, NH_2_), 7.16 (s, 1H, Ar-H), 7.21 (d, 1H,*J* = 7.2 Hz, Ar-H), 7.40–7.42 (m, 1H, Ar-H), 7.47–7.50 (m, 2H, Ar-H), 7.55 (d, 2H, *J* = 7.0 Hz, Ar-H), 7.60 (dd, 1H, *J_1_* = 7.2 Hz, *J_2_* = 3.2 Hz, Ar-H), 7.66 (dd, 1H, *J_1_* = 7.2 Hz, *J_2_* = 2.4 Hz, Ar-H), 8.36–8.38 (m, 1H, Ar-H), 8.45(d, 1H, *J* = 3.0 Hz, Ar-H), 8.79 (m, 1H, Ar-H), 9.16 (d, 1H, *J* = 1.2 Hz, Ar-H), 10.42 (s, 1H, CONH). HRMS (ESI) calculated for C_22_H_19_N_4_O_2_S [M+H]^+^: 403.1223, found: 403.1225.

*N-(3-(2-Aminothiazol-4-yl)-4-(benzyloxy)phenyl)-2-phenylacetamide* (**3g**). Yellow solid, yield 70%, m.p.: 190–193 °C. IR (KBr) 3394, 3194, 3037, 1651, 1502 cm^−1^. ^1^H-NMR (DMSO-*d_6_*): δ 3.60 (s, 2H, CH_2_), 5.20 (s, 2H, CH_2_), 6.95 (s, 2H, NH_2_), 7.10–7.12 (m, 2H, Ar-H), 7.26–7.29 (m, 1H, Ar-H), 7.31–7.35 (m, 5H, Ar-H), 7.42–7.44 (m, 2H, Ar-H), 7.46–7.50 (m, 3H, Ar-H), 8.21 (d, 1H, *J* = 2.0 Hz, Ar-H), 10.16 (s, 1H, CONH). HRMS (ESI) calculated for C_24_H_22_N_3_O_2_S [M+H]^+^: 416.1427, found: 416.1433.

*N-(3-(2-Aminothiazol-4-yl)-4-(benzyloxy)phenyl)-3-phenylpropanamide* (**3h**). Yellow solid, yield 71%, m.p.: 192–193 °C. IR (KBr) 3301, 3210, 3030, 1647, 1511 cm^−1^. ^1^H-NMR (DMSO-*d_6_*): δ 2.58 (t, 2H, *J* = 8.0 Hz, CH_2_), 2.91 (t, 2H, *J* = 8.0 Hz, CH_2_), 5.19 (s, 2H, CH_2_), 6.96 (s, 2H, NH_2_), 7.17–7.19 (m, 2H, Ar-H), 7.23–7.25 (m, 1H, Ar-H), 7.29–7.34 (m, 6H, Ar-H), 7.40–7.42 (m, 2H, Ar-H), 7.47–7.51 (m, 2H, Ar-H), 8.15 (d, 1H, *J* = 2.0 Hz, Ar-H), 9.67 (s, 1H, CONH). HRMS (ESI) calculated for C_25_H_24_N_3_O_2_S [M+H]^+^: 430.1584, found: 430.1578.

*N-(3-(2-Aminothiazol-4-yl)-4-(benzyloxy)phenyl)-2-(naphthalen-1-yl)acetamide* (**3i**). Yellow solid, yield 81%, m.p.: 246–247 °C. IR (KBr) 3375, 3183, 3022, 1651, 1625, 1513 cm^−1^. ^1^H-NMR (DMSO-*d_6_*): δ 4.11 (s, 2H, CH_2_), 5.19 (s, 2H, CH_2_), 7.06 (s, 1H, Ar-H), 7.14–7.15 (m, 1H, Ar-H), 7.31–7.33 (m, 1H, Ar-H), 7.39–7.45 (m, 4H, Ar-H), 7.47–7.49 (m, 2H, Ar-H), 7.56–7.58 (m, 3H, Ar-H), 7.72 (bs, 2H, NH_2_), 7.84 (d, 1H, *J* = 6.0 Hz, Ar-H), 7.94 (d, 1H, *J* = 6.0 Hz, Ar-H), 8.05–8.07 (m, 1H, Ar-H), 8.16 (d, 1H, *J* = 6.4 Hz, Ar-H), 10.27 (s, 1H, CONH). ^13^C-NMR (DMSO-*d*_6_): δ 169.35, 151.77, 137.11, 133.83, 133.18, 132.91, 132.45, 128.99, 128.90, 128.40, 127.96, 127.72, 126.57, 126.18, 126.02, 124.75, 120.76, 114.39, 106.22, 70.61, 40.96. HRMS (ESI) calculated for C_28_H_24_N_3_O_2_S [M+H]^+^: 466.1584, found: 466.1581.

#### 3.2.4. General Procedure for the Synthesis of *N*-(3-(2-Aminooxazol-4-yl)-4-(benzyloxy)phenyl) amides **4a–e**

The mixture of bromoacetophenone derivative **12a**–**e** (0.2 mmol) and urea (0.22 mmol) in DMF (4 mL) was refluxed until **12a**–**e** disappeared (TLC monitoring). The reaction mixture was poured into water (80 mL) and extracted with EtOAc three times (20 mL each), the organic layer was collected and washed with brine, dried over Na_2_SO_4_, evaporated *in vacuo* to dryness. The residue was purified by silica gel chromatography eluting with PE-EtOAc-Et_3_N (20:20:1) to afford **4a**–**e**.

*N-(3-(2-Aminooxazol-4-yl)-4-(benzyloxy)phenyl)benzamide* (**4a**). Pale yellow solid, yield 32%, m.p.: 173–176 °C. IR (KBr) 3299, 3196, 3042, 1643, 1498 cm^−1^. ^1^H-NMR (DMSO-*d_6_*): δ 5.30 (s, 2H, CH_2_), 6.69 (s, 2H, NH_2_), 7.15 (d, 1H, *J* = 7.2 Hz, Ar-H), 7.51–7.65 (m, 10H, Ar-H), 8.02 (d, 2H,*J* = 5.6 Hz, Ar-H), 8.36 (d, 1H, *J* = 2.0 Hz, Ar-H), 10.21 (s, 1H, CONH). HRMS (ESI) calculated for C_23_H_20_N_3_O_3_ [M+H]^+^: 386.1499, found: 386.1489.

*N-(3-(2-Aminooxazol-4-yl)-4-(benzyloxy)phenyl)-4-chlorobenzamide* (**4b**). Yellow solid, yield 37%, m.p.: 206–208 °C. IR (KBr) 3329, 3207, 3064, 1663, 1508 cm^−1^. ^1^H-NMR (DMSO-*d_6_*): δ 5.29 (s, 2H, CH_2_), 6.68 (s, 2H, NH_2_), 7.18 (d, 1H, *J* = 7.2 Hz, Ar-H), 7.41–7.43 (m, 1H, Ar-H), 7.48–7.50 (m, 2H, Ar-H), 7.55 (d, 2H, *J* = 6.4 Hz, Ar-H), 7.62–7.65 (m, 4H, Ar-H), 8.06 (d, 2H, *J* = 6.8 Hz, Ar-H), 8.34 (d, 1H, *J* = 2.0 Hz, Ar-H), 10.28 (s, 1H, CONH). HRMS (ESI) calculated for C_23_H_19_ClN_3_O_3_ [M+H]^+^: 420.1109, found: 420.1108.

*N-(3-(2-Aminooxazol-4-yl)-4-(benzyloxy)phenyl)-4-(trifluoromethyl)benzamide* (**4c**). Yellow solid, yield 30%, m.p.: 225–227 °C. IR (KBr) 3292, 3199, 3042, 1651, 1510 cm^−1^. ^1^H-NMR (DMSO-*d_6_*): δ 5.25 (s, 2H, CH_2_), 6.64 (s, 2H, NH_2_), 7.14 (d, 1H, *J* = 7.2 Hz, Ar-H), 7.36–7.39 (m, 1H, Ar-H), 7.43–7.46 (m, 2H, Ar-H), 7.50 (d, 2H, *J* = 6.0 Hz, Ar-H), 7.58–7.62 (m, 2H, Ar-H), 7.92 (d, 2H, *J* = 6.8 Hz, Ar-H), 8.17 (d, 2H, *J* = 6.8 Hz, Ar-H), 8.31 (d, 1H, *J* = 2.0 Hz, Ar-H), 10.38 (s, 1H, CONH). HRMS (ESI) calculated for C_24_H_19_F_3_N_3_O_3_ [M+H]^+^: 454.1373, found: 454.1378.

*N-(3-(2-Aminooxazol-4-yl)-4-(benzyloxy)phenyl)-4-methoxybenzamide* (**4d**). Yellow solid, yield 35%, m.p.: 185–187 °C. IR (KBr) 3329, 3241, 3052, 1654, 1521 cm^−1^. ^1^H-NMR (DMSO-*d_6_*): δ 3.86 (s, 3H, CH_3_), 5.25 (s, 2H, CH_2_), 6.78 (s, 2H, NH_2_), 7.15 (d, 1H, *J* = 7.2 Hz, Ar-H), 7.30–7.35 (m, 3H, Ar-H), 7.40–7.42 (m, 2H, Ar-H), 7.53 (d, 2H, *J* = 6.4 Hz, Ar-H), 7.58–7.66 (m, 2H, Ar-H), 8.09 (d, 2H, *J* = 7.2 Hz, Ar-H), 8.32 (d, 1H, *J* = 2.0 Hz, Ar-H), 10.31 (s, 1H, CONH). HRMS (ESI) calculated for C_24_H_22_N_3_O_4_ [M+H]^+^: 416.1605, found: 416.1600.

*N-(3-(2-Aminooxazol-4-yl)-4-(benzyloxy)phenyl)-1-naphthamide* (**4e**). Yellow solid, yield 30%, m.p.: 195–197 °C. IR (KBr) 3295, 3203, 3024, 1644, 1498 cm^−1^. ^1^H-NMR (DMSO-*d_6_*): δ 5.20 (s, 2H, CH_2_), 6.89 (s, 2H, NH_2_), 7.15 (d, 1H, *J* = 7.2 Hz, Ar-H), 7.39–7.42 (m, 1H, Ar-H), 7.45–7.47 (m, 2H, Ar-H), 7.64 (d, 2H,*J* = 6.0 Hz, Ar-H), 7.72–7.74 (m, 2H, Ar-H), 7.78–7.80 (m, 2H, Ar-H), 7.86 (d, 1H, *J* = 5.6 Hz, Ar-H), 7.93 (dd, 1H, *J_1_* = 6.8 Hz, *J_2_* = 2.4 Hz, Ar-H), 8.09–8.11 (m, 1H, Ar-H), 8.24 (d, 1H, *J* = 6.4 Hz, Ar-H), 8.38 (d, 1H, *J* = 6.4 Hz, Ar-H), 8.58 (d, 1H, *J* = 2.0 Hz, Ar-H), 10.24 (s, 1H, CONH). HRMS (ESI) calculated for C_27_H_22_N_3_O_3_ [M+H]^+^: 436.1656, found: 436.1652.

#### 3.2.5. General Procedure for the Synthesis of *N*-(3-(2-Amino-6H-1,3,4-thiadiazin-5-yl)-4-(benzyloxy) phenyl)amide hydrobromides **5a–e**.

The mixture of bromoacetophenone derivative **12a**–**e** (0.2 mmol) and hydrazine carbothioamide (0.22 mmol) in EtOH (4 mL) was refluxed for 4–6 h until **12a**–**e** disappeared (TLC monitoring). Then, HBr (0.5 mL) was added to the mixture and refluxed for 0.5 h. The formed yellow solid was filtered and recrystallized from EtOH to afford **5a**–**e**.

*N-(3-(2-Amino-6H-1,3,4-thiadiazin-5-yl)-4-(benzyloxy)phenyl)benzamide hydrobromide* (**5a**). Yellow solid, yield 53%, m.p.: 246–247 °C. IR (KBr) 3325, 3207, 3025, 1671, 1495 cm^−1^. ^1^H-NMR (DMSO-*d_6_*): δ 3.47 (s, 2H, CH_2_), 5.18 (s, 2H, CH_2_), 7.22 (d, 1H, *J* = 7.2 Hz, Ar-H), 7.35–7.38 (m, 1H, Ar-H), 7.42–7.45 (m, 2H, Ar-H), 7.48 (d, 2H, *J* = 7.2 Hz, Ar-H), 7.51–7.54 (m, 2H, Ar-H), 7.58–7.60 (m, 1H, Ar-H), 7.87 (dd, 1H, *J_1_* = 7.2 Hz, *J_2_* = 2.4 Hz, Ar-H), 7.90 (d, 1H, *J* = 6.8 Hz, Ar-H), 7.98 (d, 2H, *J* = 7.6 Hz, Ar-H), 9.10 (brs, 1H, NH_2_), 9.96 (brs, 1H, NH_2_), 10.21 (s, 1H, CONH), 13.21 (s, 1H, HBr). ^13^C-NMR (DMSO-*d*_6_): δ 165.77, 164.90, 153.08, 152.82, 135.88, 135.07, 133.20, 133.14, 132.13, 130.35, 129.00, 128.90, 128.07, 124.94, 123.79, 122.36, 113.98, 69.97, 25.39. HRMS (ESI) calculated for C_23_H_21_N_4_O_2_S [M+H]^+^: 417.1380, found: 417.1386.

*N-(3-(2-Amino-6H-1,3,4-thiadiazin-5-yl)-4-(benzyloxy)phenyl)-4-chlorobenzamide hydrobromide* (**5b**). Yellow solid, yield 48%, m.p.: 217–218 °C. IR (KBr) 3363, 3219, 3064, 1663, 1494 cm^−1^. ^1^H-NMR (DMSO-*d_6_*): δ 4.13 (s, 2H, CH_2_), 5.22 (s, 2H, CH_2_), 7.32 (d, 1H, *J* = 7.6 Hz, Ar-H), 7.37 (m, 1H, Ar-H), 7.42 (m, 2H, Ar-H), 7.50 (d, 2H, *J* = 5.6 Hz, Ar-H), 7.60–7.62 (m, 2H, Ar-H), 7.84 (dd, 1H, *J_1_* = 7.2 Hz, *J_2_* = 2.4 Hz, Ar-H), 7.97–7.99 (m, 2H, Ar-H), 8.02 (d, 1H, *J* = 2.0 Hz, Ar-H), 9.15 (brs, 1H, NH_2_), 9.92 (brs, 1H, NH_2_), 10.33 (s, 1H, CONH), 13.28 (s, 1H, HBr). ^13^C-NMR (DMSO-*d*_6_): δ 164.92, 164.63, 153.37, 152.81, 136.94, 136.83, 133.76, 132.90, 130.04, 129.01, 128.98, 128.55, 128.33, 125.03, 123.77, 122.43, 114.02, 70.81, 25.41. HRMS (ESI) calculated for C_23_H_20_ClN_4_O_2_S [M+H]^+^: 451.0990, found: 451.0993.

*N-(3-(2-Amino-6H-1,3,4-thiadiazin-5-yl)-4-(benzyloxy)phenyl)-4-(trifluoromethyl)-benzamide hydro-bromide* (**5c**). Yellow solid, yield 43%, m.p.: 222–225 °C. IR (KBr) 3315, 3198, 3025, 1671, 1503 cm^−1^. ^1^H-NMR (DMSO-*d_6_*): δ 4.15 (s, 2H, CH_2_), 5.27 (s, 2H, CH_2_), 7.23 (d, 1H, *J* = 7.2 Hz, Ar-H), 7.32–7.35 (m, 1H, Ar-H), 7.38–7.41 (m, 2H, Ar-H), 7.49 (d, 2H, *J* = 6.4 Hz, Ar-H), 7.91 (d, 2H, *J* = 6.8 Hz, Ar-H), 8.15 (d, 2H, *J* = 6.4 Hz, Ar-H), 8.45 (s, 1H, Ar-H), 9.45 (brs, 2H, NH_2_), 10.43 (s, 1H, CONH), 13.29 (s, 1H, HBr). HRMS (ESI) calculated for C_24_H_20_F_3_N_4_O_2_S [M+H]^+^: 485.1254, found: 485.1256.

*N-(3-(2-Amino-6H-1,3,4-thiadiazin-5-yl)-4-(benzyloxy)phenyl)-4-methoxybenzamide hydrobromide* (**5d**). Yellow solid, yield 40%, m.p.: 181–182 °C. IR (KBr) 3329, 3233, 3033, 1606, 1505 cm^−1^. ^1^H-NMR (DMSO-*d_6_*): δ 3.88 (s, 3H, CH_3_), 4.18 (s, 2H, CH_2_), 5.26 (s, 2H, CH_2_), 7.11 (d, 2H, *J* = 7.2 Hz, Ar-H), 7.35 (d, 1H, *J* = 8.0 Hz, Ar-H), 7.38–7.40 (m, 1H, Ar-H), 7.43–7.46 (m, 2H, Ar-H), 7.54 (d, 2H, *J* = 5.6 Hz, Ar-H), 7.89 (dd, 1H, *J_1_* = 7.2 Hz, *J_2_* = 2.4 Hz, Ar-H), 8.00 (m, 2H, Ar-H), 8.06 (d, 1H, *J* = 2.4 Hz, Ar-H), 9.20 (brs, 1H, NH_2_), 9.95 (brs, 1H, NH_2_), 10.16 (s, 1H, CONH), 13.32 (s, 1H, HBr). ^13^C-NMR (DMSO-*d*_6_): δ 165.13, 164.92, 162.40, 153.10, 152.90, 136.88, 133.31, 130.00, 129.01, 128.53, 128.32, 127.81, 124.92, 123.71, 122.34, 115.68, 114.10, 70.79, 55.92, 25.44. HRMS (ESI) calculated for C_24_H_23_N_4_O_3_S [M+H]^+^: 447.1485, found: 447.1482.

*N-(3-(2-Amino-6H-1,3,4-thiadiazin-5-yl)-4-(benzyloxy)phenyl)-1-naphthamide hydrobromide* (**5e**). Yellow solid, yield 50%, m.p.: 219–221 °C. IR (KBr) 3323, 3190, 3021, 1661, 1513 cm^−1^. ^1^H-NMR (DMSO-*d_6_*): δ 4.15 (s, 2H, CH_2_), 5.24 (s, 2H, CH_2_), 7.31 (d, 1H, *J* = 7.2 Hz, Ar-H), 7.34–7.36 (m, 1H, Ar-H), 7.38–7.42 (m, 2H, Ar-H), 7.49 (d, 2H, *J* = 5.6 Hz, Ar-H), 7.58–7.62 (m, 3H, Ar-H), 7.73 (d, 1H, *J* = 5.6 Hz, Ar-H), 7.80 (dd, 1H, *J_1_* = 6.8 Hz, *J_2_* = 2.0 Hz, Ar-H), 8.00–8.03 (m, 1H, Ar-H), 8.09 (d, 1H, *J* = 6.4 Hz, Ar-H), 8.14 (d, 1H, *J* = 2.0 Hz, Ar-H), 8.16–8.18 (m, 1H, Ar-H), 9.20 (brs, 1H, NH_2_), 9.92 (brs, 1H, NH_2_), 10.58 (s, 1H, CONH), 13.29 (s, 1H, HBr). HRMS (ESI) calculated for C_27_H_23_N_4_O_2_S [M+H]^+^: 467.1536, found: 467.1536.

### 3.3. *In Vitro* BACE-1 Inhibitory Activity Screening

All synthesized compounds were tested for their BACE 1 inhibitor activities using a fluorescence resonance energy transfer (FRET) assay, which used purified insect-expressed BACE-1 and a specific substrate. An excitation wavelength of 355 nm and an emission wavelength of 460 nm were used to monitor the hydrolysis of substrate. Compounds with inhibitory rates above 50% at 20 μg/mL were tested for IC_50_ values.

### 3.4. *In Vitro* Blood-Brain Barrier Permeability

Madin-Darby canine kidney cell line (MDCK) was obtained from Peking Union Medical College (Beijing, China). The MDR1-transfected MDCK-MDR1 cells were established in Prof. Su Zeng’s laboratory as follows: MDCK cells were seeded onto six-well plates with a seeding density of 1 × 10^5^ cells/well and cultured for 48 h. pcDNA3.1(+)/MDR1 plasmid vector was transfected into MDCK cells using Lipofectamine ^TM^ 2000 reagent according to the manufacturer’s instructions. Cells were subcultured in DMEM containing 600 µg/mL G418 for 96 h, and then replaced by DMEM containing 800 μg/mL G418 for a further 24 h. Cells were transferred to culture bottle and incubated in DMEM supplemented with 600 μg/mL G418 for 20 days. 19 stable transfected monoclonals grown on 96-well plates were obtained after dilution screening. Cells were cultured in DMEM with 10% fetal bovine serum, and grown in a humidified atmosphere of 5% CO_2_ at 37 °C. A solution of 0.25% trypsin-EDTA was used to detach the cells from flasks.

#### 3.4.1. Bidirectional Transport Studies

MDCK and MDCK-MDR1 cells were washed twice and equilibrated at 37 °C for 30 min with pre-warmed transport buffer. Hanks’ balanced salted solution (HBSS) containing HEPES (25 mM, pH 7.4). The transport buffer containing drug passed through a 0.2 μm membrane filter for degerming. For the absorption study (Apical to Basolateral), 0.5 mL incubation medium containing drug was added to the apical side as a donor chamber, 1.5 mL fresh incubation medium was added to the basolateral side as a receiver chamber. For the secretion study (Basolateral to Apical), 1.5 mL incubation medium containing drug was added to the basolateral side as a donor chamber, 0.5 mL fresh incubation medium was added to the apical side as a receiver chamber. Transport studies were conducted at 37 °C in a humidiﬁed incubator with shaking (50 rpm) for 1 h, and then the collected samples were analyzed by HPLC. Apparent permeability coefficients (P_app(A-B)_, P_app(B-A)_) and efflux ratio (P_ratio_ = (P_app(B-A)_/P_app(A-B)_) were used to evaluate the permeability and absorption profiles of compounds. P _ratio_ is an important parameter to denote if a compound is a substrate of P-gp or not.

## 4. Conclusions

Three series of 3-(2-aminoheterocycle)-4-benzyloxyphenylbenzamide derivatives were designed, based on the binding mode between aminoheterocyclic derivatives and BACE-1, synthesized and evaluated as BACE-1 inhibitors. The results showed that most of these compounds demonstrated promising BACE-1 inhibitory activities and a preliminary SAR study revealed that a 2-amino-6*H*-1,3,4-thiadizine moiety and α-naphthyl group were favorable for BACE-1 inhibition, which was supported by a molecular docking study of **5a** with BACE-1. Compound **5e** exhibited the most potent BACE-1 inhibitor activity, with an IC_50_ value of 9.9 μΜ, and also displayed favorable blood-brain barrier permeability in the MDCK and MDCK-MDR1 monolayer cell model. Our work revealed that the 2-amino-6*H*-1,3,4-thiadizine-4-benzyloxyphenylbenzamide would be a promising structural template for the development of BACE-1 inhibitors.
